# Acute Onset of Hypersomnolence and Aphasia Secondary to an Artery of Percheron Infarct and a Proposed Emergency Room Evaluation

**DOI:** 10.1155/2019/1260865

**Published:** 2019-04-08

**Authors:** Tamra Ranasinghe, SoHyun Boo, Amelia Adcock

**Affiliations:** ^1^Neurology Department, West Virginia University, USA; ^2^Radiology Department, West Virginia University, USA

## Abstract

Artery of Percheron (AOP) is a rare anatomical variant, which supplies bilateral paramedian thalami and the rostral mesencephalon via a single dominant thalamic perforating artery arising from the P1 segment of a posterior cerebral artery. AOP infarcts can present with a plethora of neurological symptoms: altered mental status, memory impairment, hypersomnolence, coma, aphasia, and vertical gaze palsy. Given the lack of classic stroke signs, majority of AOP infarcts are not diagnosed in the emergency setting. Timely diagnosis of an acute bilateral thalamic infarct can be challenging, and this case report highlights the uncommon neurological presentation of AOP infarction. The therapeutic time window to administer IV tPA can be missed due to this delay in diagnosis, resulting in poor clinical outcomes. To initiate appropriate acute ischemic stroke management, we propose a comprehensive radiological evaluation in the emergency room for patients with a high suspicion of an AOP infarction.

## 1. Introduction

Artery of Percheron (AOP) is a rare anatomical variant, which supplies bilateral paramedian thalami and the rostral mesencephalon via a single dominant thalamic perforating artery arising from the P1 segment of a Posterior Cerebral Artery (PCA) [1]. Percheron described three anatomic variations of the arterial supply to the paramedian thalamic-mesencephalic region [2]. Exact prevalence of AOP is unknown; it is estimated to be seen in 0.6% of cases of all ischemic strokes in an ischemic stroke registry of 2,750 [3]. Bilateral thalamic infarcts can present with a plethora of neurological symptom: altered mental status, memory impairment, hypersomnolence, coma, aphasia, and vertical gaze palsy [4–7].

Given the lack of classic stroke signs, majority of AOP infarcts are not diagnosed in the emergency setting. The diagnosis is usually made following a MRI brain scan, which is usually obtained outside the therapeutic window for IV tissue plasminogen activator (tPA) administration. Timely diagnosis of an acute bilateral thalamic infarct can be challenging, and this case report highlights the noncommon neurological presentation of AOP infarction. We propose a comprehensive evaluation pathway which includes an extensive diagnostic radiological approach for patients with a high suspicion of an AOP infarction. The proposed evaluation pathway needs to conclude in the emergency department in a time sensitive manner.

## 2. Case Presentation

69-year-old female with no significant past medical history with the exception of anxiety presented as a transfer from an outside hospital with acute onset of hypersomnolence and aphasia. She was last seen normal the night before by her family. Her vitals on arrival were within normal limits; blood pressure was 134/64 mmHg, heart rate was 88 per minute, respiratory rate was of 22 breaths per minutes, and she was afebrile. On exam she appeared drowsy, nonverbal, and intermittently following one-step commands. Her cranial nerves were intact and on motor exam she had mild generalized weakness but was able to move all extremities against gravity. Sensory exam was confounded by her decreased mental status. Bilateral plantar reflexes were equivocal. National Institute of Health Stroke Scale (NIHSS) was 10. She was out of the 4.5-hour time window to consider IV thrombolysis therapy and on exam her presenting symptoms did not localize to one cerebral vascular territory.

Initial diagnostic work-up: serum white blood cell count 11000/uL, hemoglobin 14.2g/dL, platelets 190000/uL, sodium 143mmol/L, potassium 5.7mmol/ (repeat 4.4mmol/L), blood urea nitrogen 34mg/dL, creatinine 1.05mg/dL, glucose 323mg/dL, troponins <7ng/L, aspartate aminotransferase 46 U/L, and alanine aminotransferase 45 U/L. Urinary analysis was positive for moderate leukocytes and negative nitrites, and her toxicology screen was negative.

**Figure 1:**
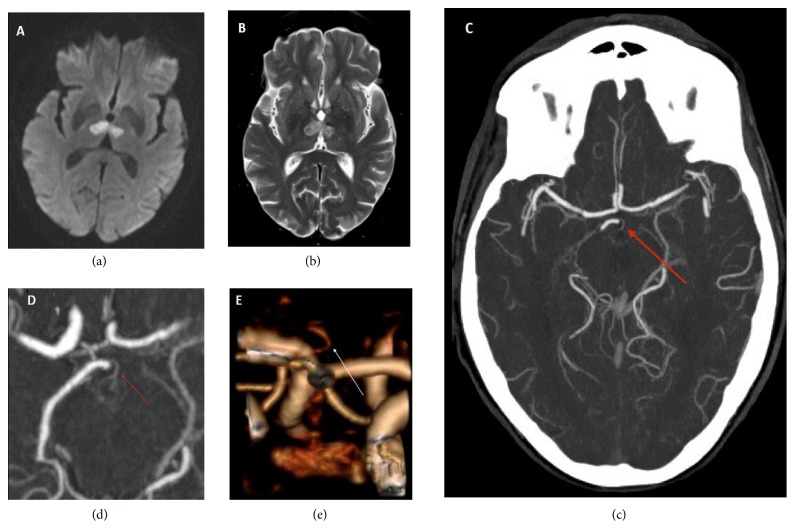
MRI brain diffusion weighted imaging series (a) and T2 (b) demonstrating bilateral paramedian thalamic infarcts. CTA vessel study. (c) Axial-Maximum Intensity Projection (MIP); (d) axial-3D-MIP; and (e) reconstructed 3D image demonstrates an Artery of Percheron (arrow) arising from the right Posterior Cerebral Artery P1 segment.

Noncontrasted CT brain demonstrated bilateral thalamic hypodensities. A CT angiogram (CTA) demonstrated focal areas of basilar artery narrowing, an Artery of Percheron (AOP) arising from the right PCA (Figures 1(c), 1(d), and 1(e)) and no large vessel occlusions. MRI brain demonstrated bilateral paramedian thalamic infarcts (Figures 1(a) and 1(b)) extending into the midbrain on diffusion weighted imaging (DWI). Her ejection fraction was 65% with no atrial septum shunt on transthoracic echocardiogram.

Her serum low density lipoprotein was 130mg/dL and her glycosylated hemoglobin was 13.8%. She was diagnosed with diabetes mellitus type 2. Her stroke etiology was thought to be secondary to small vessel disease given the arterial bed involved and her uncovered lipohyalinosis risk factors. Patient was discharged on atorvastatin 40 mg, aspirin 81 mg, and an insulin regimen. On discharge to rehab her NIHSS improved to four.

## 3. Discussion

Vascular supply to the thalamus is described as anterior territory supplied by tuberothalamic (polar) artery, the paramedian territory supplied by the thalamosubthalamic (paramedian) artery, inferolateral territory supplied by thalamogeniculate (inferolateral) artery, and the posterior territory supplied by the posterior choroidal artery [8]. The exact supply to each territory can vary due to anatomical differences and the size of the adjacent vascular territories [9]. AOP is a rare anatomical variant, which supplies bilateral paramedian thalami and the rostral mesencephalon via a single dominant thalamic perforating artery arising from the P1 segment of a PCA.

**Figure 2:**
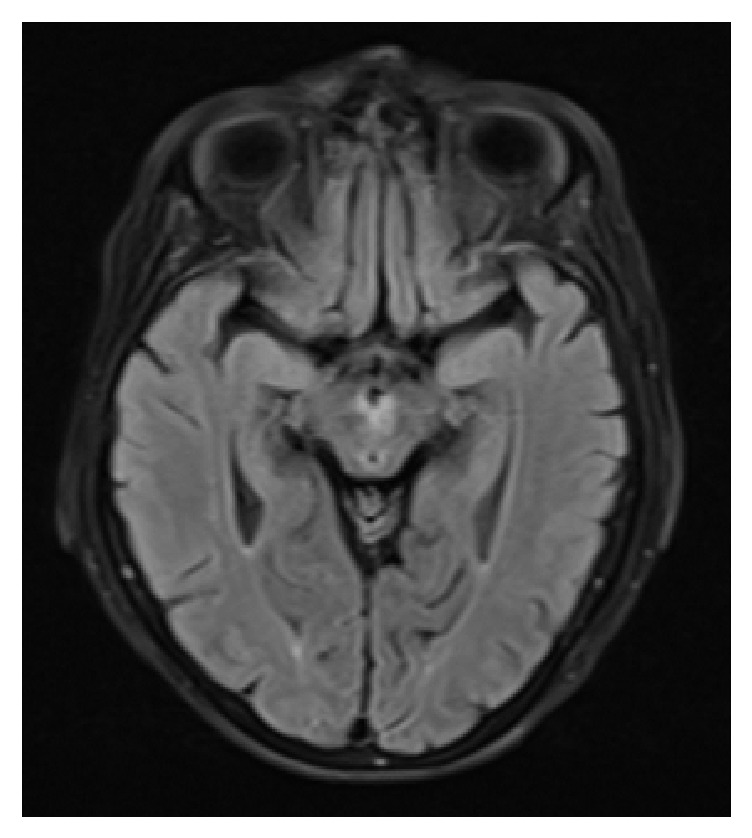
MRI brain with fluid attenuated inversion recovery (FLAIR) series demonstrating the “V sign” hyperintensity along the pial surface of the interpeduncular fossa in the midbrain.

Lazzaro N.A. et al. described four distinct patterns of AOP infarctions in their retrospective analysis of 37 patients with AOP infarcts [10]. They reported 43% with bilateral paramedian thalami with rostral midbrain, 38% with bilateral paramedian thalami without midbrain, 14% with bilateral paramedian and anterior thalami with midbrain, and 5% with bilateral paramedian and anterior thalami without midbrain infarctions. Further they described a “V-shaped hyperintensity” along the pial surface of the midbrain in the interpeduncular fossa on the axial fluid attenuated inversion recovery (FLAIR) and DWI images. The sensitivity of the “V sign” in their study group was 67% in patients with midbrain involvement. In our patient with the AOP infarction, bilateral paramedian thalami with rostral midbrain was affected with a positive “V sign” (Figure 2) seen on MRI brain FLAIR series. The AOP is rarely visualized with conventional cerebral angiogram and only four authors have successfully demonstrated this variant on conventional cerebral angiogram [10]. Therefore conventional cerebral angiograms should not be used routinely to diagnose Percheron artery occlusion. AOP is thought to be too small to be visualized on computed tomography (CT) angiogram. However, we report a very rare instance where AOP was visualized on CT angiogram (Figures 1(c), 1(d), and 1(e)).

It is vital to consider bilateral thalamic infarctions in the differential diagnosis when evaluating patients with acute onset of vague nonlateralizing neurological symptoms such as altered mental status, memory impairments, hypersomnolence, coma, aphasia, and oculomotor disturbances. A basilar tip occlusion, venous occlusion, intracranial hemorrhage, expanding subdural hematomas, subclinical seizures, Wernicke encephalopathy, neoplasms, infections, and toxic, metabolic, and inflammatory causes should be also considered in the differential diagnosis.

When a patient with nonlateralizing acute neurological symptoms presents to the emergency department, a CT brain and a CTA with perfusions should be considered with the initial blood and urine test. If the severity of the clinical features does not correlate with the CT brain findings (no acute intracranial abnormalities including hemorrhage) and the initial metabolic, toxic, and infectious work-up is negative, a bithalamic infarction should be high on the differential diagnosis. AOP infarcts are often missed on the initial CT brain scan.

**Figure 3:**
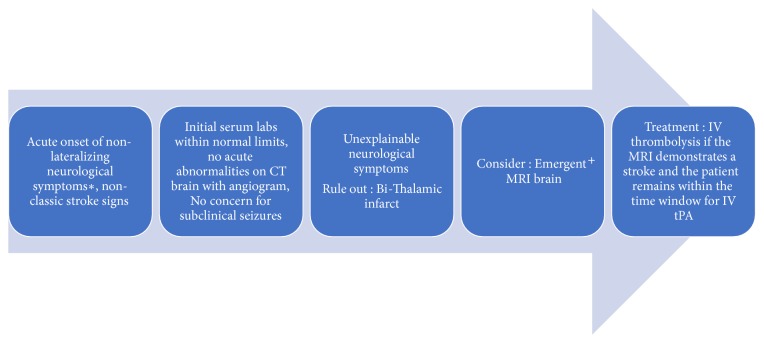
Proposed comprehensive radiological evaluation pathway to be completed in the Emergency Department. *∗*Acute onset of any of the following symptoms: altered mental status, memory impairment, hypersomnolence, coma, aphasia, and vertical gaze palsy. ^+^Emergent MRI brain without contrast with limited series of images to be done only if the patient is within the therapeutic window for thrombolysis (<4.5hrs since symptom onset).

In such patients, we propose a comprehensive evaluation pathway (Figure 3) to be completed in emergency room. This includes a CTA with perfusions and an emergent MRI brain scan if the patient is within the therapeutic time window for thrombolysis (<4.5 hours of from symptom onset). The emergent noncontrasted MRI brain scan should be limited to DWI, apparent diffusion coefficient (ADC) and gradient echo sequences (GRE) series. Given the limited series, MRI brain scan should be completed in a time sensitive manner ~15-20 minutes. If the MRI brain is positive, implying acute ischemia and the patient remains within the therapeutic window for thrombolysis, IV tPA should be administered. Maintaining a high suspicion for thalamic infarct, with AOP occlusion as one etiology, and a low threshold for MRI in patients presenting acutely with otherwise unexplainable neurological symptoms may facilitate diagnosis and decrease morbidity [9].

However, it is essential to keep in mind the low risk of complications including symptomatic intracranial hemorrhage in stroke mimics receiving IV tPA [11, 12]. Hence, if there is a high suspicion of an acute ischemic stroke, CT brain is negative for hemorrhage, and the initial work-up is not suggestive of an alternate diagnosis, thrombolysis with IV tPA should not be delayed to conduct further imaging.

## 4. Conclusion

Thalamic pathology should be considered in patients with vague nonlateralizing neurological symptoms. Diagnosis of Percheron artery infarction is challenging and often made later in presentation due to lack of clinical awareness and the nonclassic stroke signs/symptoms on presentation. The therapeutic time window to administer IV tPA can be missed due to this delay in diagnosis, resulting in poor clinical outcomes. To initiate appropriate acute ischemic stroke management, we propose a comprehensive radiological evaluation in the emergency room for patients with a high suspicion of an AOP infarction.
